# Predicting time to treatment in follicular lymphoma on watchful waiting using baseline metabolic tumour burden

**DOI:** 10.1007/s00432-022-04138-3

**Published:** 2022-07-02

**Authors:** Lucia Leccisotti, Daria Maccora, Rosalia Malafronte, Francesco D’Alò, Elena Maiolo, Salvatore Annunziata, Vittoria Rufini, Alessandro Giordano, Stefan Hohaus

**Affiliations:** 1 Unit of Nuclear Medicine, Department of Diagnostic Imaging, Radiation Oncology and Haematology, Fondazione Policlinico Universitario Agostino Gemelli IRCCS, Largo A. Gemelli, 8, 00168 Rome, Italy; 2 University Department of Radiological Sciences and Haematology, Università Cattolica del Sacro Cuore, Rome, Italy; 3 Unit of Extramedullary Lymphoproliferative Diseases, Fondazione Policlinico Universitario Agostino Gemelli IRCCS, Rome, Italy

**Keywords:** Follicular lymphoma, PET, PET/CT, FDG, Metabolic tumour volume, Watchful waiting

## Abstract

**Purpose:**

Asymptomatic patients with follicular lymphoma (FL) and a low tumour burden can be followed without initial therapy, a strategy called watchful waiting (WW). Prediction of the time to treatment (TTT) is still a challenge. We investigated the prognostic value of baseline total metabolic tumour volume (TMTV) and whole-body total lesion glycolysis (WB-TLG) to predict TTT in patients with FL on WW.

**Methods:**

We conducted a retrospective study of 54 patients with FL (grade 1–3a) diagnosed between June 2013 and December 2019, staged with FDG PET/CT, and managed on WW. Median age was 62 years (range 34–85), stage was advanced (III–IV) in 57%, and FLIPI score was intermediate to high (≥ 2) in 52% of the patients.

**Results:**

The median TMTV and WB-TLG were 7.1 and 43.3, respectively. With a median follow-up of 59 months, 41% of patients started immuno-chemotherapy. The optimal cut-points to identify patients with TTT within 24 months were 14 for TMTV (AUC 0.70; 95% CI 51–88) and 64 for WB-TLG (AUC 0.71; 95% CI 52–89) (*p* < 0.005). The probability of not having started treatment within 24 months was 87% for TMTV < 14 and 53% for TMTV ≥ 14 (*p* < 0.005). TMTV was independent of the FLIPI score for TTT prediction. Patients with both FLIPI ≥ 2 and TMTV ≥ 14 had only an 18% probability of not having started treatment at 36 months, while this probability was 75% in patients with TMTV < 14.

**Conclusion:**

Metabolic tumour volume parameters may add information to clinical scores to better predict TTT and better stratify patients for interventional studies.

## Introduction

Follicular lymphoma (FL) represents the second most common non-Hodgkin lymphoma subtype and the most frequent indolent lymphoma in Western countries (Freedman 2015; Ekberg et al. 2020). FL is a biologically heterogeneous disease with median survival times exceeding 10 years, but prognosis varies widely among individuals. The broad spectrum of clinical behaviour of FL ranges from cases undergoing spontaneous remission to those with adverse characteristics and an aggressive clinical course (Link et al. 2013). For many affected patients who have low tumour burden and are asymptomatic, a watchful waiting (WW) approach is recommended, starting chemotherapy only after the onset of symptoms (Brice et al. 1997). However, approximately 25–40% of FL patients managed with WW develop progression of disease within 2 years. Prognostic models, as the Follicular Lymphoma International Prognostic Index (FLIPI) and the most recently described FLIPI2 (Solal-Céligny et al. 2004; Federico et al. 2009) only poorly identify FL patients at risk for early progression during WW (Solal-Céligny et al. 2012). In the era of precision medicine, there is a growing interest in patient risk stratification at diagnosis using biomarkers that would allow for a prospective definition of different risk groups. This approach would offer the chance of defining a treatment tailored to the individual risk profile. Currently, fluorine-18-fluorodeoxy-glucose positron emission tomography/computed tomography (FDG PET/CT) is recommended for staging and response assessment in all FDG-avid lymphomas, including FL (Barrington et al. 2014; Cheson et al. 2014). We reasoned that functional parameters derived from staging FDG PET/CT could help to identify the subgroup of FL patients on initial WW with a high risk of progression within 2 years. The total metabolic tumour volume (TMTV), a quantitative parameter representing the total volume of all FDG-avid lesions, has been reported as a functional parameter able to predict the patient outcome at diagnosis in high tumour burden FL (Meignan et al. 2016). Our study aims to investigate the prognostic value of baseline TMTV and whole-body total lesion glycolysis (WB-TLG) in low tumour burden FL patients on WW and their added value to existing clinical prognostic indices.

## Materials and methods

### Subjects

We performed a retrospective analysis on low tumour burden FL patients referred to the Haematology Unit of Fondazione Policlinico Universitario Agostino Gemelli IRCCS between June 2013 and December 2019. Inclusion criteria were age ≥ 18 years, histologically confirmed FL (grade 1–3a in accordance with the World Health Organization Classification; Sabattini et al. 2010), initial WW for low tumour burden disease according to the *Group d’Etude des Lymphomes Folliculaires* (GELF) criteria (Brice et al. 1997), availability of baseline whole-body FDG PET/CT images and at least 24 months of follow-up. Exclusion criteria were previous chemo- and/or radiotherapy as well as synchronous neoplasia. Patient management and treatment options were in accordance with good clinical practice rules. Clinical and pathological data were collected from internal medical records, including patient baseline characteristics, initial approach, and reasons for starting a systemic treatment following initial WW. This retrospective study was conducted according to the institutional ethical guidelines and in accordance with the Declaration of Helsinki. The retrospective data collection and anonymous analysis were approved by our Ethics Committee (ID 3834/2021).

### FDG PET/CT imaging and analysis

All patients fasted at least 6 h before the FDG administration. Blood glucose levels were checked to be less than 200 mg/dL before FDG injection in each patient. Whole-body PET/CT was acquired using a Biograph mCT (Siemens Healthcare) scanner, 60 ± 10 min after intravenous administration of FDG (3 MBq/kg). After scout CT acquisition, a CT transmission scan (50 mAs, 120 kV, slice thickness of 3 mm, 2.80-slice increment) was acquired from the skull base to the mid-thigh for photon attenuation correction and anatomical localization. PET imaging was acquired in three-dimensional mode, 2 min per bed position, with a 256 × 256 matrix and pixel size/slice thickness of 3.18 × 3.18/5.00 mm. After normalization and correction for dead time, randoms and scatters, PET data were reconstructed using an iterative algorithm (ordered-subsets expectation maximization, 2 iterations and 21 subsets), with the combined effect of point spread function (PSF) modelling and time of flight (TOF). PET/CT images were transferred to a commercially available multimodality reading solution with molecular imaging applications for oncology. Two experienced nuclear physicians (LL and DM), who were blinded to patient clinical characteristics and outcomes, retrospectively reviewed all FDG PET/CT scans. Images were evaluated by visual assessment and quantitative analysis. Volumes of interest (VOIs) were segmented using an automatic whole-body segmentation (LesionID^®^, MIM Software Inc., Cleveland, OH, USA; Werner-Wasik et al. 2012). The contours of the hypermetabolic lesions were automatically created using the liver as the standard reference point. Particularly, the Positron Emission Tomography Response Criteria in Solid Tumours (PERCIST) value was used as the threshold to identify hypermetabolic lesions (nodal and extranodal) and calculated using the following formula: PERCIST = (1.5 × liver mean) + (2 × liver standard deviation; Major et al. 2020). All areas with normal physiologic FDG uptake (e.g., heart, brain, salivary glands, urinary system…) were manually excluded. Inter-observer reproducibility of PET measurements was high (intraclass correlation coefficient = 0.92 with 95% confidence interval [CI] 0.89–0.95). PET parameters such as SUVmax, SUVmean, MTV and TLG were extracted for each lesion. SUVmax and SUVmean are defined as the greatest uptake in a single voxel within the semi-automatically defined VOI and the average SUV throughout the VOI, respectively. MTV (cm^3^) is defined as the volume of tumour tissues with increased FDG uptake above the threshold described. TLG was calculated as the product of SUVmean and MTV. TMTV (cm^3^) and WB-TLG were calculated as the sum of MTV and TLG of all nodal and extra-nodal FDG-avid lesions.

### Statistical analysis

Time to treatment (TTT) was calculated using the dates of initial diagnosis and initiation of systemic treatment. Overall survival (OS) was calculated from the initial diagnosis to death by any cause or last follow-up. Receiver operating characteristics (ROC) curves and the corresponding area under the curve (AUC) were used to define the optimal cut-offs for TTT within 24 months for FDG PET/CT metrics. Survival curves were constructed by the Kaplan–Meier method. Prognostic significances of FDG PET/CT parameters and clinical variables were assessed by univariate analysis. Variables with significant associations were included in a multivariate analysis using the Cox proportional hazards model. A *p* value < 0.05 was considered statistically significant. All the statistical analyses used STATA 12 statistical software.

## Results

**Fig. 1:**
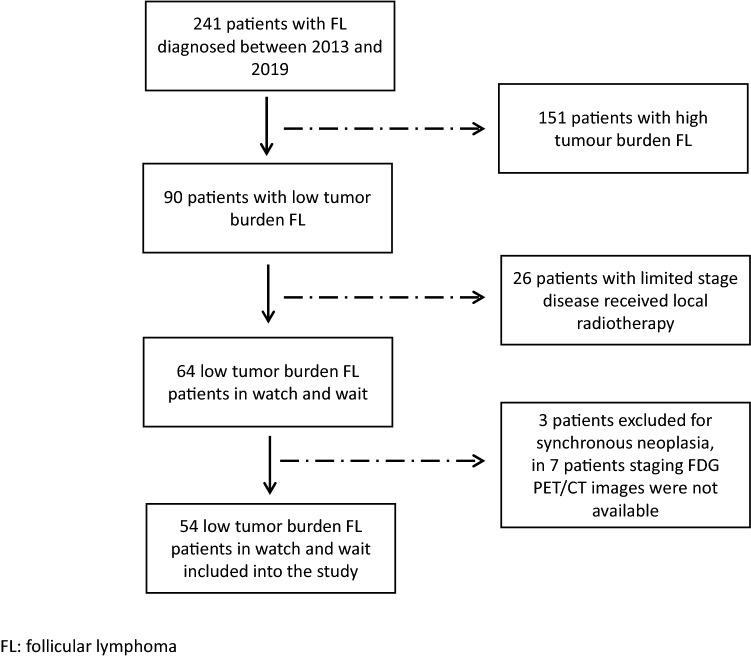
Flow chart of patient selection for the clinical study

A total of 54 patients with newly diagnosed FL who were followed on WW were identified in our institutional database of 241 patents with FL diagnosed between June 2013 and December 2019 and included in this study (Fig. 1). Median age was 62 years (range 34–85), 31 (57.4%) patients had stage III/IV disease, and 28 (51.5%) patients had an intermediate-high (≥ 2) FLIPI score. Further patient characteristics of the study population are reported in Table 1. After a median follow-up of 59 months (range 31–111), 22 (40.7%) patients started immuno-chemotherapy due to disease progression. Median TTT was 22 months (range 7–79).

**Table 1:** Baseline patient and disease characteristics of study population (*n* = 54)

Age (years) at initial diagnosis, mean ± SD (range)	61 ± 12 (34–85)
*Sex, n (%)*	
Male	28 (52)
Female	26 (48)
*Grading disease, n (%)*	
G1	6 (11)
G2	35 (65)
G3	8 (15)
Others	5 (9)
*Bone marrow involvement, n (%)*	
Yes	13 (24)
No	39 (72)
N/A	2 (4)
*Stage disease, n (%)*	
I	9 (17)
II	14 (26)
III	11 (20)
IV	20 (37)
*FLIPI, n (%)*	
0	9 (17)
1	17 (31.5)
2	17 (31.5)
3	11 (20)
*FLIPI 2, n (%)*	
0	14 (26)
1	25 (46)
2	10 (18)
3	2 (4)
4	1 (2)
N/A	2 (4)

FLIPI, Follicular Lymphoma International Prognostic Index; *N/A*, not available

### Associations between metabolic and clinical parameters

**Fig. 2:**
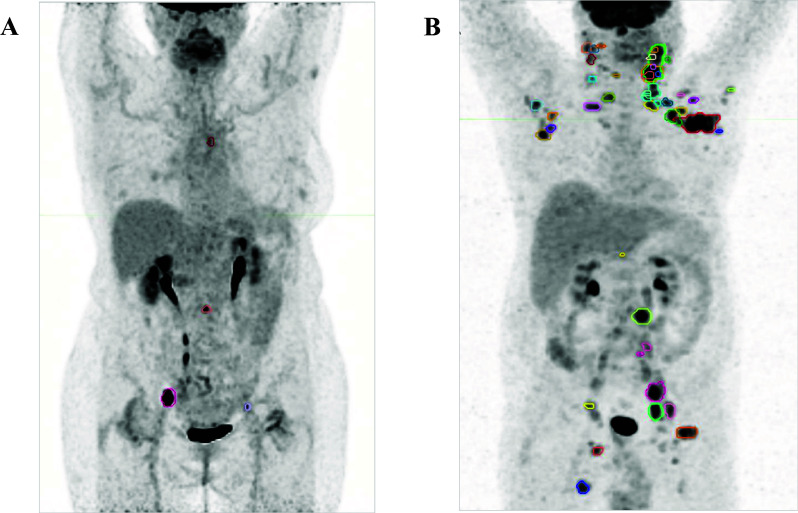
Total metabolic tumour volume (TMTV) and whole-body total lesion glycolysis (WB-TLG) delineated at baseline FDG PET/CT using an automatic whole-body segmentation software. **A.** A 68-year-old female patient with low tumour burden follicular lymphoma (FL): grading 2, stage IIA, Follicular Lymphoma International Prognostic Index (FLIPI) = 1. She had been on WW for 79 months, when she started R-COMP for disease progression. TMTV = 7.3, WB-TLG = 44.6; **B.** A 58-year-old male patient with low tumour burden FL: grading 2, stage IVA, FLIPI = 2. He started treatment with six cycles R-CHOP + 2 × Rituximab maintenance for disease progression 9.4 months after diagnosis. TMTV = 143.8 and WB-TLG = 657.1

Median values of all baseline FDG PET/CT parameters are reported in Table 2. In patients with detectable FDG uptake TMTV and WB-TLG ranged from 1.3 to 180.1, and from 3.8 to 1080.3, respectively. Two representative cases of patients with low and high metabolic burdens are shown in Fig. 2.

**Table 2:** Baseline FDG PET/CT parameters

Contoured sites per patient (total no. 427)	3 (0–11)
SUVmax	7.33 (0–12.42)
SUVmean	3.51 (0–5.04)
TMTV	7.15 (0–43.74)
WB-TLG	43.35 (0–152.60)

All data are expressed as median and (interquartile range); SUVmax, maximum Standardized Uptake Value; SUVmean, mean Standardized Uptake Value; TMTV, total metabolic tumour volume; WB-TLG, whole-body total lesion glycolysis

A significant association was found between TMTV or WB-TLG and the stage of disease (*p* = 0.01), more extensive nodal disease (4 or more lymph nodes involved, *p* = 0.01) and FLIPI ≥ 2 (*p* = 0.02) (Table 3). We did not find a significant association between PET parameters and sex, age, haemoglobin, serum lactate dehydrogenase (LDH), follicular grading, disease bulk, or bone marrow involvement.

**Table 3:** Factors associated with metabolic parameters in univariate analysis

	TMTV	
	*z*	*p*
Sex	0.99	0.32
Age	− 0.93	0.35
Haemoglobin	− 0.99	0.32
Lactate Dehydrogenase	− 0.73	0.46
Follicular grading	0.82	0.41
Disease bulk	0.49	0.62
Bone marrow involvement	0.58	0.55
Stage of disease	− 2.50	**0.01**
≥ 4 lymph nodes	− 2.45	**0.01**
FLIPI ≥ 2	− 2.23	**0.02**

TMTV, total metabolic tumour volume; FLIPI, Follicular Lymphoma International Prognostic Index

A *p* value < 0.05 was considered statistically significant (in bold)

### Baseline metabolic parameters and TTT

**Fig. 3:**
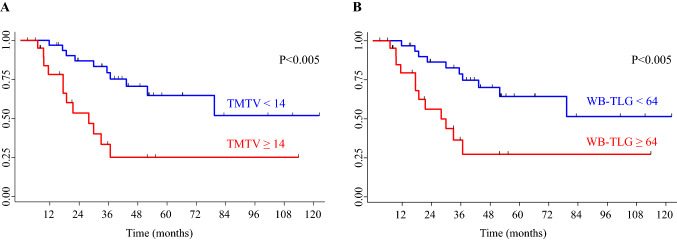
Time to treatment curves according to total metabolic tumour volume (TMTV) and whole-body total lesion glycolysis (WB-TLG) optimal cut-off values. The probability of not having started treatment at 24 months after diagnosis is 87% (95% CI 69–95) for patients with TMTV < 14 and 53% (95% CI 28–74) for patients with TMTV ≥ 14 (*p* < 0.005) (**A**), and 86% (95% CI 68–95) for patients with WB-TLG < 64 and 56% (95% CI 31–75) for patients with TWB-TLG ≥ 64 (*p* < 0.005) (**B**)

We first analysed the associations between metabolic parameters and TTT. Baseline SUVmax and SUVmean were not associated with TTT, while TMTV and WB-TLG were significantly associated with TTT. Using a ROC analysis, we found an optimal cut-off value for TTT within 24 months of 14 for TMTV (sensitivity 67%, specificity 75%, AUC 0.70, 95% CI 51–88) and of 64 for WB-TLG (sensitivity 67%, specificity 72%, AUC 0.71, 95% CI 52–89). Using these cut-offs, the probability of not having started treatment at 24 months after diagnosis was 87% (95% CI 69–95) for patients with TMTV < 14 and 53% (95% CI 28–74) for patients with TMTV ≥ 14 (*p* < 0.005) (Fig. 3A), and was 86% (95% CI 68–95) for patients with WB-TLG < 64 and 56% (95% CI 31–75) for patients with TWB-TLG ≥ 64 (*p* < 0.005) (Fig. 3B). When restricting the analysis to 31 patients with advanced stage (III to IV) the prognostic impact of TMTV and WB-TLG was confirmed.

### Clinical parameters and TTT

**Fig. 4:**
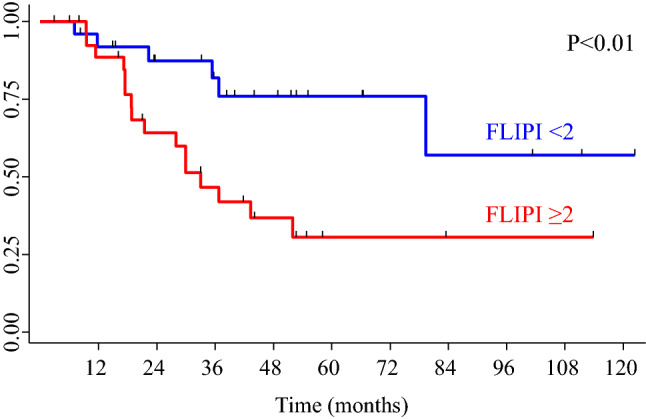
Time to treatment (TTT) curves according to Follicular Lymphoma International Prognostic Index (FLIPI) Score (< 2 and ≥ 2). FLIPI ≥ 2 resulted significantly associated to inferior TTT (*p* < 0.01)

In univariate analysis, FLIPI ≥ 2 was significantly associated with inferior TTT (*p* < 0.01) (Fig. 4). The expected probability of not starting treatment at 24 months after diagnosis was 87% (95% CI 65–96) for FL patients with FLIPI < 2 and 64% (95% CI 42–79) for patients with FLIPI ≥ 2. Bone marrow involvement, bulky mass, involvement of more than 4 nodal areas, LDH and beta2-microglobulin were not associated to TTT.

### Combining metabolic parameters and clinical parameters

**Fig. 5:**
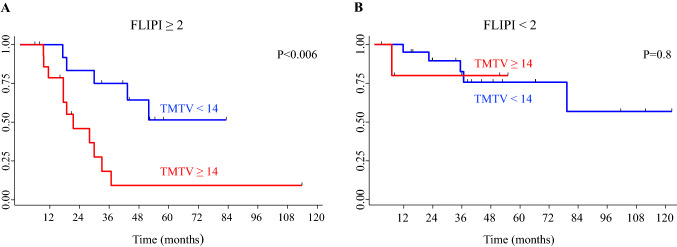
Time to treatment (TTT) curves in (**A**) patients with intermediate-high (≥ 2) Follicular Lymphoma International Prognostic Index (FLIPI) and (**B**) in patients with FLIPI < 2 according to the total metabolic tumour volume (TMTV) cut-off of 14. **A** Follicular lymphoma patients with both TMTV ≥ 14 and intermediate-high FLIPI (≥ 2) have only an 18% probability of not having starting treatment at 36 months (95% CI 3–44) while patients with TMTV < 14 and FLIPI ≥ 2 have 75% probability of not starting treatment at 36 months (95% CI 41–91). **B** TMTV could not discriminate the risk of TTT in patients with a low FLIPI score (< 2)

PET parameters were entered into a multivariate analysis with clinical variables and FLIPI: TMTV and WB-TLG resulted significantly related to TTT (*p* < 0.05) (Tables 4, 5). We also analysed the prognostic role of TMTV and WB-TLG combined with the prognostic score FLIPI. We found that FL patients with both TMTV ≥ 14 and intermediate-high (≥ 2) FLIPI scores had only an 18% probability of not having started treatment at 36 months (95% CI 3–44) while patients with TMTV < 14 and FLIPI ≥ 2 had 75% probability of not starting treatment at 36 months (95% CI 41–91) (Fig. 5A), while TMTV could not discriminate the risk of TTT in patients with a low FLIPI score (0, 1) (Fig. 5B). Similar results were found when combining WB-TLG to FLIPI ≥ 2.

**Table 4:** Multivariate analysis of time to treatment including TMTV and FLIPI

Parameter	HR	(95% CI)	*p*
TMTV ≥ 14 versus < 14	2.9	1.2–7.1	0.002
FLIPI ≥ 2 versus < 2	2.4	0.9–6.4	0.07

HR, hazard ratio; CI, confidence interval; TMTV, total metabolic tumour volume; FLIPI, Follicular Lymphoma International Prognostic Index

**Table 5:** Multivariate analysis of time to treatment including WB-TLG and FLIPI

Parameter	HR	(95% CI)	*p*
WB-TLG ≥ 64 versus < 64	2.6	1.1–6.4	0.03
FLIPI ≥ 2 versus < 2	2.4	0.9–6.4	0.07

HR, hazard ratio; CI, confidence interval; WB-TLG, whole-body total lesion glycolysis; FLIPI, Follicular Lymphoma International Prognostic Index

## Discussion

In our retrospective study, we found that baseline TMTV and WB-TLG are independent prognostic factors of time to start treatment in patients with low tumour burden FL on initial WW. Additionally, baseline TMTV and WB-TLG can improve risk-stratification by conventional prognostic indices as the FLIPI score. Our data suggest that combining information from clinical data and PET/CT at diagnosis could be helpful to identify a subgroup of patients who will require treatment within a short time.

Our data fit well into the scenario of the use of TMTV and WB-TLG for prognostication in lymphomas (Esfahani et al. 2013; Sasanelli et al. 2014; Cottereau et al. 2016a, b; Cottereau et al. 2017). Large retrospective analyses of prospective trials show that baseline TMTV measurement performs better than clinical and molecular indices and can complement them for improving risk stratification (Meignan et al. 2021). We found that SUV was not predictive for TTT. This is in line with other studies on the predictive values of PET parameters indicating that TMTV and WB-TLG are more reproducible and potentially more accurate quantitative predictors of prognosis at baseline and response to treatment in lymphomas (Meignan et al. 2016, 2021). TMTV has been identified as a prognostic parameter in high tumour burden FL (Meignan et al. 2016). A pooled analysis of 185 patients with high tumour burden FL reported that a baseline TMTV ≥ 510 is associated with an increased risk for poor outcome (Meignan et al. 2016). As expected, TMTV was significantly lower in our low-tumour burden patients with FL indicating that our patients had indeed a low tumour burden and were candidates for a WW strategy. We identified baseline TMTV of 14 and WB-TLG of 64 as optimal cut-points for TTT. The combination of PET/CT and FLIPI scores stratified the population into two risk categories. Patients with TMTV (or WB-TLG) higher than cut-off and intermediate to high FLIPI score (≥ 2) had a high risk to initiate treatment early during WW strategy, with an only 18% probability of not having started treatment at 36 months. Conversely, FL patients with low TMTV and FLIPI ≥ 2 had a 75% probability of not starting treatment at 36 months. Similarly, combining these 2 parameters has been reported to result in better risk stratification in high tumour burden FL (Meignan et al. 2016). In the study of Meignan et al., high TMTV and intermediate-high FLIPI2 scores resulted associated with a 5-year progression-free survival (PFS) of 20%, high TMTV or intermediate-high FLIPI2 score with 5-year PFS of 46%, and low TMTV and low FLIPI2 with 5-year PFS of 69% (Meignan et al. 2016). In addition, the combination of TMTV and FLIPI2 score identified patients at high risk of early progression following therapy. In another study from the LYSA group, high pre-treatment TMTV combined with a positive end of induction therapy identified a subgroup of high tumour burden FL patients with increased risk of death and only 23% 5-year PFS (Cottereau et al. 2018). A study reporting a retrospective analysis of 84 high tumour burden FL found baseline WB-TLG as an independent prognostic factor for PFS and OS (Zhou et al. 2019).

To our knowledge, our data demonstrate for the first time the predictive role of TMTV and WB-TLG in a population of low tumour burden FL on initial WW. Tumour burden is defined by several parameters that are surrogates for high tumour burden (e.g., tumour mass with a diameter larger than 7 cm, the involvement of at least three nodal areas each of which with a diameter > 3 cm). Since almost all lymphoma lesions are FDG avid, the total volume of PET-positive lesions, the TMTV, is highly correlated with the total tumour burden. The use of TMTV and WB-TLG could be a promising supplement to classify low and high tumour burden. TMTV and WB-TLG are easily measurable by commercially available software. Patients with FL often have numerous lesions of various sizes and sometimes not homogeneous FDG uptake which could represent a challenge for measurement of TMTV and WB-TLG. The modern softwares allow to obtain volume computation in a few seconds and only leaves the exclusion of non-pathological regions which have been erroneously selected by the software as a task to the operator. As a result, TMTV measurement could become now possible in clinical practice. Optimal cut-off points for TMTV differ significantly between studies (Cottereau et al. 2017; Im et al. 2018; Burggraaff et al. 2020; Meignan et al. 2021). Reasons for these differences consist of variations in the study population, tumour characteristics and the segmentation method used. Different segmentation methods will result in different volumes depending on the SUV of the lesions. However, the same method, even when using different softwares, if the patient population is similar should result in a similar median TMTV. In previous studies, TMTV and/or WB-TLG have been usually measured by applying fixed absolute SUV threshold, such as 2, 2.5 or a fixed relative threshold of 40–41% (Meignan et al. 2014, 2016; Sasanelli et al. 2014; Boellaard et al. 2015; Cottereau et al. 2016a). We applied an innovative and highly reproducible thresholding method based on the PERCIST criteria including background relative thresholds. It has been reported to be more accurate than methods that use absolute or fixed percentage thresholds (Im et al. 2018). The latter could overestimate the volume of the lesions with low SUVmax that are frequent in FL or include the volume of non-tumour regions located between small nodes with high uptake. Similarly, a background relative threshold method has been used successfully in the GOYA study including more than a thousand diffuse large B-cell lymphoma patients (Kostakoglu et al. 2021). Further harmonization in the procedure of segmentation is expected to solve this issue in the future. Nevertheless, TMTV measured at baseline in FDG-avid lymphomas has been associated with prognosis regardless of the segmentation method used, with similar predictive performance when different methods were compared in the same patient population (Ilyas et al. 2019).

There are some limitations to this study. This was a single-centre retrospective study, in which a limited number of patients were analysed. We chose TTT as outcome parameter, as this is objective and simple to be determined. As all patients were followed in our centre, the decision to start therapy that could be different between centres was more homogeneous. The good prognosis of patients with FL did not allow for analysis of overall survival. In fact, all patients were alive at the time of the last follow-up. Further prospective studies including a higher number of patients will be needed to confirm the threshold values we identified for TMTV and WB-TLG before this information can be used to implement interventional clinical studies for low-tumour burden patients with a high risk for an early need to start therapy during WW.

## Conclusions

TMTV and WB-TLG recorded at staging FDG PET/CT in low-tumour burden FL can identify patients at high risk for early disease progression and a short time to start treatment during WW. In addition, the combination of quantitative PET parameters with conventional prognostic indices such as FLIPI score may contribute to develop risk-adapted individualized care in FL patients.

## Data Availability

The datasets generated during and/or analysed during the current study are available from the corresponding author on reasonable request.
